# Effects of rAAV-Mediated Overexpression of *sox9* and TGF-*ß* via Alginate Hydrogel-Guided Vector Delivery on the Chondroreparative Activities of Human Bone Marrow-Derived Mesenchymal Stromal Cells

**DOI:** 10.1155/2023/4495697

**Published:** 2023-08-18

**Authors:** Wei Liu, Jagadeesh K. Venkatesan, Mahnaz Amini, Tamás Oláh, Gertrud Schmitt, Henning Madry, Magali Cucchiarini

**Affiliations:** Center of Experimental Orthopaedics, Saarland University and Saarland University Medical Center, Homburg/Saar, Germany

## Abstract

Recombinant adeno-associated virus (rAAV) vectors have a strong potential to promote the healing of traumatic cartilage defects and osteoarthritic lesions upon delivery and overexpression of therapeutic genes from suitable biomaterials that support a controlled release of the candidate constructs. The goal of the present work is to examine whether the administration of chondrogenic rAAV *sox9* and rAAV TGF-*ß* gene vehicles via alginate hydrogel-guided vector delivery stimulates the biological and chondroreparative activities of human bone marrow-derived mesenchymal stromal cells (hMSCs) as a source of improved reparative cells for future implantation in sites of cartilage damage. The delivery of rAAV using an alginate (AlgPH155) hydrogel system is successfully achieved in hMSCs over time (21 days), leading to the effective overexpression of *sox9* and TGF-*ß* that significantly increases the proliferation and chondrogenic differentiation activities of the cells relative to control (marker *lacZ*) gene transfer while advantageously preventing premature hypertrophy, osteogenesis, and mineralization. This study reveals the potential of alginate hydrogel-based systems to control the delivery of rAAV (*sox9* and TGF-*ß*) gene vectors to adeptly trigger the chondroreparative activities of hMSCs for future applications that aim at improving cartilage repair.

## 1. Introduction

The articular cartilage, the gliding avascular tissue with a highly specialized composition and structure that covers the extremities of the articulating bones in joints, has a limited ability for regeneration [1, 2]. Articular cartilage injuries are common problems that remain challenging in the clinics as none of the currently applied treatments restore the original hyaline cartilage tissue in sites of cartilage lesions, with a complete structural and functional integrity [2–4]. Novel, targeted approaches are therefore critically needed to improve the quality of the repair tissue in cartilage lesions, and biological treatments may provide strong tools to trigger the intrinsic reparative mechanisms underlying cartilage repair. In this regard, the cartilage-specific sex-determining region Y-type high mobility box 9 transcription factor (SOX9) [5, 6] and the transforming growth factor beta (TGF-*ß*) [7, 8] represent attractive biological cues as highly potent agents capable of promoting cartilage repair by enhancing the proliferation of chondrocytes and the production of extracellular matrix (ECM) components, while preventing cartilage degradation [5–12]. However, a direct application of therapeutic SOX9 and TGF-*ß* in their recombinant forms remains hindered by their short pharmacological half-life [13, 14].

Gene transfer is a promising strategy to achieve effective and durable articular repair as it is based on the introduction of foreign gene sequences in a target cell population that may be expressed over prolonged periods of time, especially when delivered via viral gene vectors [13, 14]. Recombinant gene vehicles based on the nonpathogenic human adeno-associated (AAV) parvovirus (rAAV vectors) have notable advantages over other families of gene vehicles, including their relatively low immunogenicity and toxicity, their high and persistent gene transfer efficiencies due to their maintenance as steady episomes that can stably express the carried transgenes (up to 100% efficiencies for months to years) [15], and their capacity to target both dividing and nondividing cells that make them highly preferred systems, in particular for chondroreparation purposes [13, 14]. Yet, an adapted application of rAAV vectors in the clinics is still restricted by possible immune responses in the recipient, including a preexistence of neutralizing antibodies against the AAV capsid proteins in the human population [16].

To address this issue, the controlled release of rAAV vectors from biomaterials represents a workable strategy to enhance the spatial and temporal availability of therapeutic gene sequences (and products) in a defined target [17]. Alginate (AlgPH155), based on a polysaccharide from brown algae, is a promising biocompatible material that can easily form gels under straightforward conditions (room temperature and normal atmospheric pressure) to encapsulate reparative cells, recombinant factors as external stimuli, and gene transfer vectors for therapeutic delivery in cartilage lesions [18]. Their ideal biocompatibility attributes together with a lack of immunogenicity and of inflammatory responses make it a promising candidate for various tissue engineering approaches [18], especially as an experimentally controlled release platform.

The goal of the present study was to target human bone marrow-derived mesenchymal stromal cells (hMSCs) as a source of chondroreparative cells for cartilage repair using rAAV vectors coding for the highly chondrogenic *sox9* and TGF-*ß* via release from an alginate (AlgPH155)-based hydrogel system in order to improve the biological activities of these cells for future approaches of implantation in sites of cartilage damage (Supplementery material: Table of contents) in light of our previous work showing that such a hydrogel system can effectively target hMSCs when delivering a reporter rAAV gene vector (*lacZ* gene coding for *E. coli β*-galactosidase) via effective controlled rAAV vector release for up to 21 days [19]. The data show that the successful rAAV-mediated overexpression of *sox9* and TGF-*ß* via AlgPH155-guided gene vector application (rAAV-h*sox9*/AlgPH155, rAAV-hTGF-*ß*/AlgPH155) significantly and safely enhanced the adapted chondrogenesis of hMSCs over time (21 days as the standard time point of optimal MSC chondrogenesis) [7] relative to control (rAAV-*lacZ*/AlgPH155) treatment while reducing undesirable hypertrophy, osteogenesis, and mineralization. These findings show the potential of the current rAAV (*sox9*, TGF-*ß*)/alginate hydrogel system for the future treatment of articular cartilage lesions in patients.

## 2. Materials and Methods

### 2.1. Reagents

All reagents were purchased from Sigma (Munich, Germany) unless otherwise indicated. Sodium alginate (GRINDSTED AlgPH155, molecular weight = 140 kDa, mannuronic to glucuronic (M : G) ratio = 1 : 5, viscosity = 350–550 mPass) was purchased from Danisco (Copenhagen, Denmark). The anti-SOX9 (C-20) and anti-TGF-ß (V) antibodies were from Santa Cruz Biotechnology (Heidelberg, Germany), the anti-type-II collagen (AF-5710) and anti-type-I collagen (AF-5610) antibodies from Acris (Hiddenhausen, Germany), and the anti-type-X collagen (COL-10) antibody from Sigma. The biotinylated secondary antibodies and ABC reagent were from Vector Laboratories (Alexis Deutschland GmbH, Grünberg, Germany). The AAVanced concentration reagent was from System Bioscience (Heidelberg, Germany), the Quantikine enzyme-linked immunosorbent assay (ELISA) from R&D Systems (Wiesbaden, Germany), and the *β*-gal staining kit and cell proliferation reagent WST-1 from Roche Applied Science (Mannheim, Germany).

### 2.2. Human Bone Marrow-Derived Mesenchymal Stromal Cells

The study was approved by the Ethics Committee of the Saarland Physicians Council (ethics application approval registration number: Ha67/12), and all procedures were performed in accordance with the Helsinki Declaration. In addition, all patients gave informed consent before being enrolled in the study. Human bone marrow aspirates (∼15 ml; 0.4–1.2 × 10^9^ cells/ml) were prepared from the distal femurs of patients undergoing total knee arthroplasty (*n* = 3, age: 71 ± 10 years) [20] at the Department of Orthopaedics Surgery of the Saarland University Medical Center (Homburg/Saar, Germany). Human bone marrow-derived mesenchymal stromal cells (hMSCs) were isolated from human bone marrow aspirates by washing and centrifuging the human bone marrow aspirates in Dulbecco's modified Eagle medium (DMEM) and by resuspending the pellets in red blood cell lysis buffer with DMEM (1 : 1) [19, 20]. The mixtures were washed and resuspended in DMEM 10% fetal bovine serum (FBS), 100 U/ml penicillin G, and 100 *μ*l/ml streptomycin (growth medium) for cell plating and maintenance in T75 flasks at 37°C under 5% CO_2_. A medium change was performed after 24 h using the growth medium and the recombinant basic fibroblast growth factor (FGF-2; 1 ng/ml) for expansion without effects on the cell potency [21], followed by changes every 2-3 days and replating when cells reach a density of 85%, using cells at no more than passage 1-2 [19]. Cells were used for further treatments at the denoted cell densities.

### 2.3. Preparation of Plasmids and rAAV Vectors

The constructs were derived from pSSV9, an AAV-2 genomic clone [22, 23]. rAAV-*lacZ* carries the *E. coli β*-galactosidase (*lacZ*) reporter gene, rAAV-hTGF-*ß* a 1.2-kb human transforming growth factor beta 1 (hTGF-*ß*) cDNA fragment, and rAAV-h*sox9* a FLAG-tagged human *sox9* cDNA, all under the control of the cytomegalovirus immediate-early (CMV-IE) promoter [24]. The vectors were packaged as conventional (not self-complementary) vectors using a helper-free, two-plasmid transfection system in 293 cells with the packaging plasmid pXX2 and the adenovirus helper plasmid pXX6 [24]. The vector preparations were purified by extensive dialysis and titrated by RT-PCR, averaging 10^10^ transgene copies/ml (∼1/500 functional recombinant viral particles) [24].

### 2.4. Incorporation of rAAV Vectors in Alginate Hydrogel

**Figure 1:**
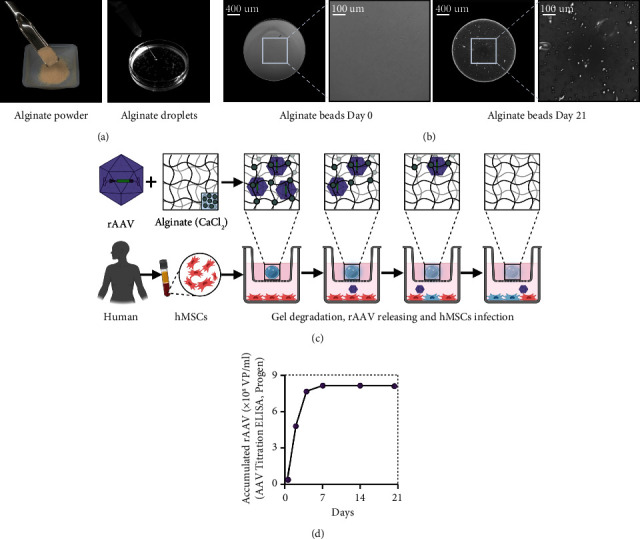
Design of the rAAV/alginate hydrogel system to trigger chondroreparative hMSCs. (a) Preparation of the alginate (AlgPH155) powder and droplets. (b) Physical properties of the alginate hydrogel system as an over time delivery system of rAAV vectors (light microscopy; magnification ×4; scale bars: 400 *μ*m/100 *μ*m). (c) Formulation and release of rAAV vectors from the alginate hydrogel system to target hMSCs as chondroreparative cells for cartilage repair. (d) Principle of the assessment of rAAV vector-controlled release from the alginate hydrogel system [19].

The system was prepared with 0.3% alginate (AlgPH155) in PBS (Figures 1(a) and 1(b)) [19]. rAAV-loaded capsules were prepared by dropping the dispersion of polymers containing rAAV preparations (10^10^ transgene copies) to the polymeric solutions in calcium chloride (102 mM) in 6-well plates using a syringe with a needle of 27G (Figure 1(c)) [19]. Alginate crosslinking with calcium was performed at room temperature for a brief period of time (3–5 sec) [19]. The system was kept in culture in 24-well plates (Figure 1(c)). A condition without rAAV vector was not included here as it was already tested previously [19], showing no difference *versus* rAAV-*lacZ* in terms of reporter or therapeutic gene expression and of hMSC chondrogenic potency. Assessment of rAAV vector-controlled release from the current alginate hydrogel system has been previously reported by us [19] (Figure 1(d)).

### 2.5. Gene Transfer via rAAV/Alginate Hydrogel Systems

Monolayer cultures of hMSCs (2 × 10^4^ cells/well in 24-well plates) were kept for 21 days with the rAAV/alginate hydrogel systems (rAAV-h*sox9*/AlgPH155, rAAV-hTGF-*ß*/AlgPH155, or rAAV-*lacZ*/AlgPH155) (20 *μ*l per well; 10^10^ transgene copies/ml) at 37°C with medium change every 3-4 days (Figure 1(c)) [19].

### 2.6. Detection of Transgene Expression

The expression of *lacZ* was measured by X-gal staining using a *β*-gal staining kit [25]. Expression of SOX9 and TGF-*ß* was quantitatively estimated by immunocytochemistry using specific primary antibodies, biotinylated secondary antibodies, and the ABC method with diaminobenzidine as the chromogen [20, 24]. Secondary immunoglobulins were monitored without the primary antibody and then visualized via a light microscope [20, 24]. The expression of TGF-*ß* was also examined by specific ELISA. In brief, hMSCs in monolayer culture were washed twice and maintained for 24 h in a serum-free medium prior to the collection of culture medium supernatants [20, 24]. Supernatants were taken at the denoted time points and centrifuged to separate debris [20, 24]. Measurements were then carried out with a GENios spectrophotometer/fluorometer (Tecan, Crailsheim, Germany) [24].

### 2.7. Biological Evaluations

Cell proliferation was estimated using the cell proliferation reagent WST-1 by recording the absorbance at 450 nm being proportional to the cell numbers on a GENios spectrophotometer/fluorometer (Tecan), and cell proliferation was provided as a direct index [19]. Cultures were also harvested for papain digestion to evaluate the DNA contents by the Hoechst 33258 fluorometric assay, the proteoglycans contents by binding to dimethyl-methylene blue (DMMB) dye, and the total cellular protein contents for normalization by using a protein assay (Pierce Thermo Scientific Protein Assay; Thermo Fisher Scientific, Schwerte, Germany) [20, 24]. Measurements were performed on a GENios spectrophotometer/fluorometer (Tecan) [24].

### 2.8. Histological and Immunocytochemical Analyses

hMSCs in a monolayer culture were harvested and fixed in 4% formalin. Fixed cells were stained with alcian blue for glycosaminoglycans (GAGs) as previously reported with excess stain being washed off with ddH_2_O [20, 24]. The stain was quantified by overnight solubilization in 6 M guanidine hydrochloride to monitor absorbance at 595 nm [24]. Fixed cultures were also stained with hematoxylin/eosin (H&E) for cellularity, with safranin O for proteoglycans, and with alizarin red for matrix mineralization [24]. Expression of SOX9, TGF-*ß*, and type-II/-I/-X collagen was monitored by immunocytochemistry using specific primary antibodies (anti-SOX9: 1/60; anti-TGF-*ß*: 1/50; anti-type-I collagen: 1/200; anti-type-II collagen: undiluted; anti-type-X collagen: 1/200) and secondary antibodies (1 : 200) [20, 24]. To control for secondary immunoglobulins, sections were processed with omission of the primary antibody.

### 2.9. Histomorphometry

The cell densities on H&E-stained cultures (the ratio of cell numbers to the area of the site evaluated) [24] and the average optical density (AOD) of alcian blue-stained, safranin O-stained, alizarin red-stained, and of SOX9/TGF-*ß*/anti-type-II/-I/-X collagen immunostained cultures (the ratio of staining intensity to stained area of the site evaluated) were measured at four randomized sites in the cultures using the CellSens program 1.12 (Olympus, Hamburg, Germany), ImageJ 1.53K (ImageJ, Maryland, USA), and Adobe Photoshop adobe systems software CS6 (Adobe Systems, Unterschleissheim, Germany) [26]. Alcian blue staining, safranin O staining, alizarin red staining, and SOX9/TGF-*ß*/anti-type-II/-I/-X collagen immunostaining were performed (uniformity, density) using a modified Bern score grading system (0 = no staining; 1 = heterogeneous and/or weak staining; 2 = homogeneous and/or moderate staining; 3 = homogeneous and/or intense staining; 4 = very intense staining) [27]. Scoring was blindly performed by two individuals with regard to the conditions.

### 2.10. Real-Time PCR Analysis

Total cellular RNA was extracted with the RNeasy Protect Mini Kit and on-column RNase-free DNase treatment (Qiagen, Hilden, Germany) [20]. RNA was eluted in 40 *μ*l RNase-free water, and reverse transcription was performed using 8.2 *μ*l of eluate and the 1^st^ strand cDNA synthesis kit for RT-PCR (AMV) (Roche Applied Science) [20]. RT-PCR amplification was performed using 2 *μ*l of cDNA product with Brilliant SYBR Green QPCR master mix (Stratagene, Agilent Technologies, Waldbronn, Germany) on an Mx3000P QPCR system (Stratagene) [20]. The following conditions were used: 10 min at 95°C, cycles of amplification (30 sec denaturation at 95°C, 1 min annealing at 60°C, and 30 sec extension at 72°C), denaturation (1 min at 95°C), and final incubation (30 sec at 55°C) [20]. The primers (Applied Biosystems, Inchinnan, UK) employed were as follows: SOX9 (transgene and chondrogenic marker; forward 5′-ACACACAGCTCACTCGACCTTG-3′; reverse 5′-GGGAATTCTGGTTGGTCCTCT-3′), TGF-*ß* (transgene; forward5′-TACCATGCCAACTTCTGTCTGGGA-3′; reverse 5′-ATGTTGGACAACTGCTCCACCTTG-3′),type-II collagen (COL2A1; chondrogenic marker; forward 5′-GGACTTTTCTCCCCTCTCT-3′; reverse 5′-GACCCGAAGGTCTTACAGGA-3′),type-I collagen (COL1A1; osteogenic marker; forward 5′-ACGTCCTGGTGAAGTTGGTC-3′; reverse 5′-ACCAGGGAAGCCTCTCTCTC-3′),type-X collagen (COL10A1; marker of hypertrophy; forward 5′-CCCTCTTGTTAGTGCCAACC-3′; reverse 5′-AGATTCCAGTCCTTGGGTCA-3′), and glyceraldehyde-3-phosphate dehydrogenase (GAPDH; housekeeping gene and internal control; forward, 5′-GAAGGTGAAGGTCGGAGTC-3′; and reverse, 5′-GAAGATGGTGATGGGATTTC-3′) (all 150 nM final concentration) [20]. Control conditions included reactions with water and nonreverse-transcribed mRNA, and product specificity was confirmed by melting curve analysis and agarose gel electrophoresis. The threshold cycle (Ct) value for each gene was obtained for each amplification using MxPro QPCR software (Stratagene). Values were normalized to GAPDH expression using the the 2^−ΔΔCt^ method [20].

### 2.11. Statistical Analysis

Data are given as the mean and standard deviation or the median and interquartile range for each separate experiment. All conditions were performed in triplicate in three independent experiments per patient, using all the patients in all experiments. The Shapiro–Wilk normality test and the *F* test or the Brown–Forsythe test were employed to check for normal distribution and equal variance. The Wilcoxon test, the Mann–Whitney test, and the Kruskal–Wallis test were conducted for nonparametric analysis and Dunn's test for multiple comparisons. The one sample *t*-test, the unpaired *t*-test, ordinary one-way ANOVA, and the Welch test were performed for parametric analysis and Dunnett's/Dunnett's T3 tests for multiple comparisons. The Shapiro–Wilk normality test and ordinary one-way ANOVA including the Brown–Forsythe and Dunnett's tests were used for the X-gal staining AODs, while the Shapiro–Wilk normality test and the Kruskal–Wallis test including Dunn's test were used for the X-gal staining scores. The Shapiro–Wilk normality test and the Mann–Whitney test were used for the TGF-*ß* ELISA. The Shapiro–Wilk normality test and ordinary one-way ANOVA including the Brown–Forsythe and Dunnett's tests were used for the anti-SOX9, -TGF-*ß*, and -type-I/-II/-X collagen immunocytochemistry AODs, while the Shapiro–Wilk normality test and the Kruskal–Wallis test including Dunn's test were used for the anti-SOX9, -TGF-*ß*, and -type-I/-II/-X collagen immunocytochemistry scores. The Shapiro–Wilk normality test and ordinary one-way ANOVA including the Brown–Forsythe and Dunnett's tests were used for the Hoechst 33258 assay and for the analysis of the cell densities. The Shapiro–Wilk normality test and the Brown–Forsythe and Welch tests including the Brown–Forsythe and Dunnett's tests were used for the WST assay. The Shapiro–Wilk normality test and the Kruskal–Wallis test including Dunn's test were used for the alcian blue staining values and scores, for the safranin O staining scores, and for the DMMB assay. The Shapiro–Wilk normality test and ordinary one-way ANOVA including the Brown–Forsythe and Dunnett's tests were used for the alcian blue staining AODs. The Shapiro–Wilk normality test and the Brown–Forsythe and Welch tests including the Brown–Forsythe and Dunnett's tests were used for the safranin O staining AODs. The Shapiro–Wilk normality test, the Wilcoxon test, the Mann–Whitney test, the one sample *t*-test, and the unpaired *t*-test including the *F* test were used for the real-time RT-PCR analysis. The Shapiro–Wilk normality test and the Kruskal–Wallis test including Dunn's test were used for the alizarin red staining scores, while the Shapiro–Wilk normality test and ordinary one-way ANOVA including the Brown–Forsythe and Dunnett's tests were used for the alizarin red staining AODs. *P* values/adjusted *P* values were reported with *P* < 0.05 considered statistically significant. Box plot diagrams always showed the interquartile range (upper and lower borders of the boxes), the minimum and maximum (whiskers), the mean value (+), the median (middle line), and the individual data points (dots). All calculations were performed with Prism v.8.2.1 (GraphPad Software, San Diego, USA).

## 3. Results

### 3.1. Effective rAAV-Mediated *lacZ*, *sox9*, and TGF-*ß* Overexpression in hMSCs upon Alginate Hydrogel-Guided Vector Delivery

The reporter rAAV-*lacZ*/alginate (rAAV-*lacZ*/AlgPH155) hydrogel system was first tested for its ability to promote *lacZ* overexpression in hMSCs *in vitro*, relative to the candidate rAAV-h*sox9*/alginate (rAAV-h*sox9*/AlgPH155) and rAAV-hTGF-*ß*/alginate (rAAV-hTGF-*ß*/AlgPH155) hydrogel systems. The candidate rAAV-h*sox9*/AlgPH155 and rAAV-hTGF-*ß*/AlgPH155 hydrogel systems were then tested for their respective ability to promote *sox9* and TGF-*ß* overexpression in hMSCs *in vitro*, relative to control conditions including the reporter rAAV-*lacZ*/AlgPH155 hydrogel system and the counterpart system (rAAV-hTGF-*ß*/AlgPH155 or rAAV-h*sox9*/AlgPH155).

**Figure 2:**
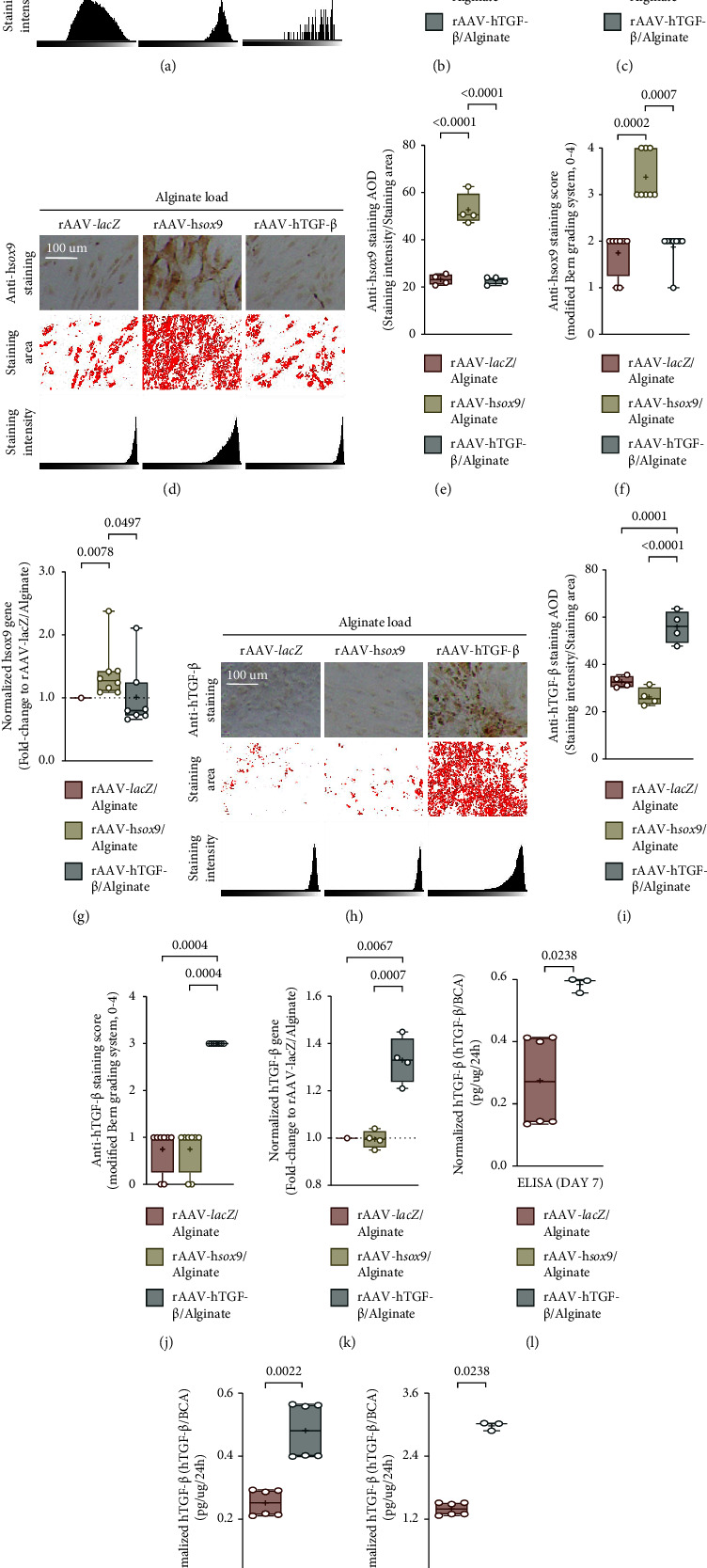
Detection of rAAV-mediated *lacZ*, *sox9*, and TGF-*ß* overexpression over time (21 days) in hMSCs upon alginate hydrogel-guided vector delivery. (a) X-gal staining with an estimation (b) of the average optical density (AOD) and (c) of the modified Bern score (magnification ×10; scale bars: 100 *μ*m). (d) SOX9 immunodetection with an estimation (e) of the AOD and (f) of the modified bern score (magnification ×10; scale bars: 100 *μ*m). (g) Analysis of *sox9* gene expression by real-time RT-PCR. (h) TGF-*ß* immunodetection with an estimation (i) of the AOD and (j) of the modified bern score (magnification ×10; scale bars: 100 *μ*m). (k) Analysis of TGF-*ß* gene expression by real-time RT-PCR. (l–n) Evaluation of the levels of TGF-*ß* expression by ELISA in the supernatants of hMSC culture after 7 (l), 14 (m), and 21 days (n).

Effective rAAV-mediated *lacZ* overexpression was observed in hMSCs after 21 days by X-gal staining, with significantly higher *lacZ* expression levels achieved when using rAAV-*lacZ*/AlgPH155 relative to rAAV-h*sox9*/AlgPH155 (up to 1.2- and more than 8-fold difference in average optical density (AOD) and score, respectively, *P* ≤ 0.0232) or to rAAV-hTGF-*ß*/AlgPH155 (up to 1.2- and 8-fold difference in AOD and score, respectively, *P* ≤ 0.04) (Figures 2(a)–2(c)).

Successful rAAV-mediated *sox9* overexpression was noted in hMSCs after 21 days by SOX9 immunodetection, with significantly higher SOX9 expression levels achieved when using rAAV-h*sox9*/AlgPH155 relative to rAAV-*lacZ*/AlgPH155 (up to 2.3- and 1.5-fold difference in AOD and score, respectively, *P* ≤ 0.0002) or to rAAV-hTGF-*ß*/AlgPH155 (up to 2.3- and 1.5-fold difference in AOD and score, respectively, *P* ≤ 0.0007) (Figures 2(d)–2(f)). These data were corroborated by the results of a real-time RT-PCR analysis, with significantly higher *sox9* gene expression levels relative to rAAV-*lacZ*/AlgPH155 or to rAAV-hTGF-*ß*/AlgPH155 (up to 1.3-and 1.6-fold difference, respectively, *P* ≤ 0.0497) (Figure 2(g)).

Similarly, successful rAAV-mediated TGF-*ß* overexpression was seen in hMSCs after 21 days by TGF-*ß* immunodetection, with significantly higher TGF-*ß* expression levels achieved when using rAAV-hTGF-*ß*/AlgPH155 relative to rAAV-*lacZ*/AlgPH155 (up to 1.7- and 3-fold difference in AOD and score, respectively, *P* ≤ 0.0004) or to rAAV-h*sox9*/AlgPH155 (up to 2.1- and 3-fold difference in AOD and score, respectively, *P* ≤ 0.0004) (Figures 2(h)–2(j)). These data were corroborated by the results of a real-time RT-PCR analysis, with significantly higher TGF-*ß* gene expression levels relative to rAAV-*lacZ*/AlgPH155 or to rAAV-h*sox9*/AlgPH155 (up to 1.3-fold difference, *P* ≤ 0.0067) (Figure 2(k)) and by TGF-*ß* ELISA, showing prolonged, significantly higher TGF-*ß* expression levels achieved over time when using rAAV-hTGF-*ß*/AlgPH155 relative to rAAV-*lacZ*/AlgPH155 (up to 2.2-, 1.9-, and 2.2-fold difference on days 7, 14, and 21, respectively, *P* ≤ 0.0238) (Figures 2(l)–2(n)).

### 3.2. Effects of rAAV-Mediated *sox9* and TGF-*ß* Overexpression on the Biological and Chondrogenic Activities of hMSCs upon Alginate Hydrogel-Guided Vector Delivery

The candidate rAAV-h*sox9*/AlgPH155 and rAAV-hTGF-*ß*/AlgPH155 hydrogel systems were next evaluated for their respective effects on the biological and chondrogenic activities of hMSCs *in vitro* relative to the control rAAV-*lacZ*/AlgPH155.

**Figure 3:**
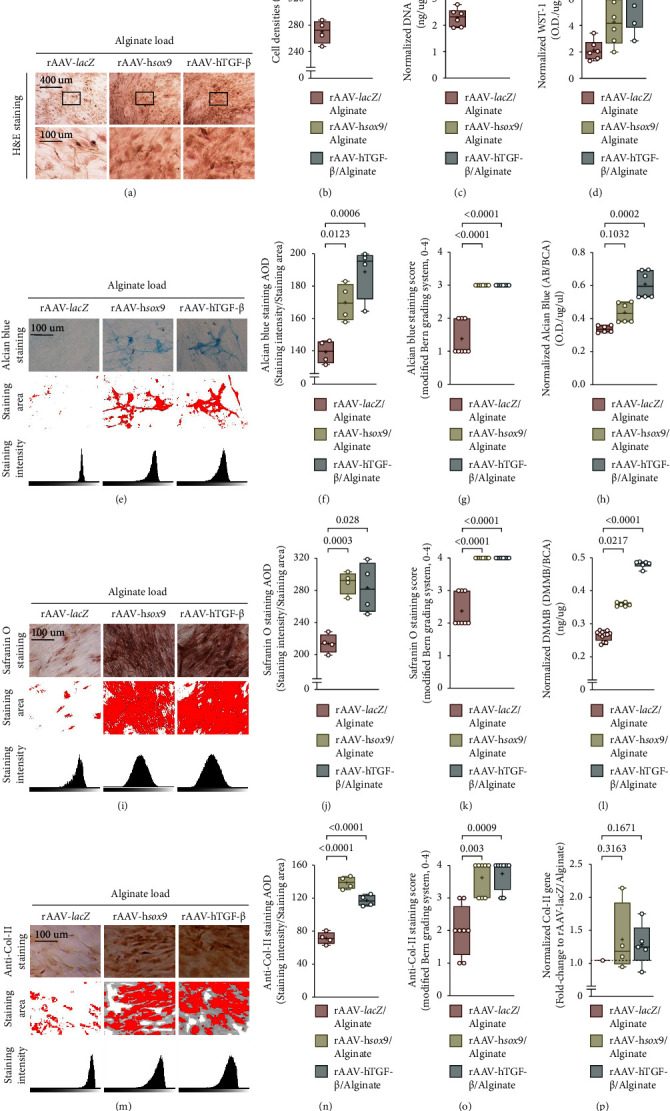
Effects of rAAV-mediated *sox9* and TGF-*ß* overexpression over time (21 days) on the biological and chondrogenic activities of hMSCs upon alginate hydrogel-guided vector delivery. (a–d) Evaluation of the cell proliferation indices (a) by H&E staining (magnification ×10; scale bars: 100 *μ*m) with an estimation (b) of the cell densities, (c) of the DNA contents (hoechst 33258 assay), and (d) of the cell proliferation indices (WST-1 assay). (e–l) Evaluation of GAG and proteoglycan deposition (e) by alcian blue staining (magnification ×10; scale bars: 100 *μ*m) with an estimation (f) of the AOD and (g) of the modified Bern score followed (h) by alcian blue staining solubilization, (i) by safranin O staining (magnification ×10; scale bars: 100 *μ*m) with an estimation (j) of the AOD and (k) of the modified Bern score and by an estimation (l) of the proteoglycan contents (DMMB assay). (m–o) Evaluation of type-II collagen deposition (m) by immunodetection (magnification ×10; scale bars: 100 *μ*m) with an estimation (n) of the AOD and (o) of the modified Bern score. (p) Analysis of type-II collagen gene expression by real-time RT-PCR.

The application of rAAV-h*sox9*/AlgPH155 or rAAV-hTGF-*ß*/AlgPH155 significantly enhanced the densities of hMSCs after 21 days relative to rAAV-*lacZ*/AlgPH155 (up to 1.4-fold difference, *P* ≤ 0.0001) as seen by H&E staining (Figures 3(a) and 3(b)). These findings were corroborated by an estimation of the DNA contents (Hoechst 33258 assay) in the cells at a similar time point (up to 1.4- and 1.6-fold difference with rAAV-h*sox9*/AlgPH155 and rAAV-hTGF-*ß*/AlgPH155 relative to rAAV-*lacZ*/AlgPH155, respectively,*P* ≤ 0.0149) (Figure 3(c)) and by the results of a WST-1 assay on the cell proliferation indices (up to 2.9-fold difference with rAAV-hTGF-*ß*/AlgPH155 relative to rAAV-*lacZ*/AlgPH155, *P*=0.0184), although statistical significance was not reached with rAAV-h*sox9*/AlgPH155 (up to 2-fold difference relative to rAAV-*lacZ*/AlgPH155, *P*=0.0554) (Figure 3(d)).

Administration of rAAV-h*sox9*/AlgPH155 or rAAV-hTGF-*ß*/AlgPH155 significantly increased the deposition of GAGs and proteoglycans in hMSCs after 21 days, relative to rAAV-*lacZ*/AlgPH155. This was first noted by alcian blue staining of the cultures (up to 1.2- and 3-fold difference in AOD and score, respectively, with rAAV-h*sox9*/AlgPH155 relative to rAAV-*lacZ*/AlgPH155, *P* ≤ 0.0123; up to 1.4- and 3-fold difference in AOD and score, respectively, with rAAV-hTGF-*ß*/AlgPH155 relative to rAAV-*lacZ*/AlgPH155, *P* ≤ 0.0006) (Figures 3(e)–3(g)) with a corroboration following alcian blue staining solubilization (up to 1.8-fold difference with rAAV-hTGF-*ß*/AlgPH155 relative to rAAV-*lacZ*/AlgPH155, *P*=0.0002), although statistical significance was not reached with rAAV-h*sox9*/AlgPH155 (up to 1.3-fold difference relative to rAAV-*lacZ*/AlgPH155, *P*=0.1032) (Figure 3(h)). This was also seen by safranin O staining of the cultures (up to 1.4- and 2-fold difference in AOD and score, respectively, with rAAV-h*sox9*/AlgPH155 relative to rAAV-*lacZ*/AlgPH155, *P* ≤ 0.0003; up to 1.3- and 2-fold difference in AOD and score, respectively, with rAAV-hTGF-*ß*/AlgPH155 relative to rAAV-*lacZ*/AlgPH155, *P* ≤ 0.028) (Figures 3(i)–3(k)). These findings were substantiated by an estimation of the proteoglycan contents (the DMMB assay) in the cells at a similar time point (up to 1.3- and 1.8-fold difference with rAAV-h*sox9*/AlgPH155 and rAAV-hTGF-*ß*/AlgPH155 relative to rAAV-*lacZ*/AlgPH155, respectively, *P* ≤ 0.0217) (Figure 3(l)).

Treatment with rAAV-h*sox9*/AlgPH155 or rAAV-hTGF-*ß*/AlgPH155 significantly enhanced the levels of type-II collagen deposition in hMSCs after 21 days, relative to rAAV-*lacZ*/AlgPH155 (up to 1.9- and 2-fold difference in AOD and score, respectively, with rAAV-h*sox9*/AlgPH155 relative to rAAV-*lacZ*/AlgPH155, *P* ≤ 0.003; up to 1.6- and 2-fold difference in AOD and score, respectively, with rAAV-hTGF-*ß*/AlgPH155 relative to rAAV-*lacZ*/AlgPH155, *P* ≤ 0.0009) as seen by type-II collagen immunodetection (Figures 3(m)–3(o)). These findings were corroborated by the result of a real-time RT-PCR analysis although statistical significance was not reached (up to 1.3- and 1.2-fold difference with rAAV-h*sox9*/AlgPH155 and rAAV-hTGF-*ß*/AlgPH155 relative to rAAV-*lacZ*/AlgPH155, respectively; *P*=0.1671 and *P*=0.3163, respectively) (Figure 3(p)).

### 3.3. Effects of rAAV-Mediated *sox9* and TGF-*ß* Overexpression on the Hypertrophy, Osteogenesis, and Mineralization of hMSCs upon Alginate Hydrogel-Guided Vector Delivery

The candidate rAAV-h*sox9*/AlgPH155 and rAAV-hTGF-*ß*/AlgPH155 hydrogel systems were finally examined for their respective effects on hMSC hypertrophy, osteogenesis, and mineralization *in vitro* relative to the control rAAV-*lacZ*/AlgPH155.

**Figure 4:**
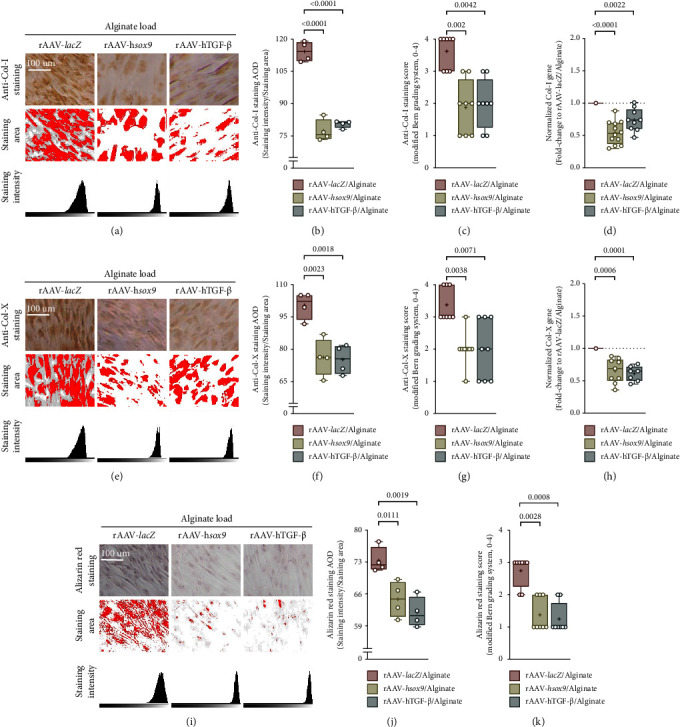
Effects of rAAV-mediated *sox9* and TGF-*ß* overexpression over time (21 days) on the osteogenesis and mineralization of hMSCs upon alginate hydrogel-guided vector delivery. (a–c) Evaluation of type-I collagen deposition (a) by immunodetection (magnification ×10; scale bars: 100 *μ*m) with an estimation (b) of the AOD and (c) of the modified Bern score. (d) Analysis of type-I collagen gene expression by real-time RT-PCR. (e–g) Evaluation of type-X collagen deposition (e) by immunodetection (magnification ×10; scale bars: 100 *μ*m) with an estimation (f) of the AOD and (g) of the modified Bern score. (h) Analysis of type-X collagen gene expression by real-time RT-PCR. (i–k) Evaluation of matrix mineralization (i) by alizarin red staining (magnification ×10; scale bars: 100 *μ*m) with an estimation (j) of the AOD and (k) of the modified Bern score.

The application of rAAV-h*sox9*/AlgPH155 or rAAV-hTGF-*ß*/AlgPH155 significantly reduced the levels of osteogenic type-I collagen deposition in hMSCs after 21 days, relative to rAAV-*lacZ*/AlgPH155 (up to 1.5- and 2-fold difference in AOD and score, respectively, with rAAV-h*sox9*/AlgPH155 relative to rAAV-*lacZ*/AlgPH155, *P* ≤ 0.002; up to 1.4- and 2-fold difference in AOD and score, respectively, with rAAV-hTGF-*ß*/AlgPH155 relative to rAAV-*lacZ*/AlgPH155, *P* ≤ 0.0042) as seen by type-I collagen immunodetection (Figures 4(a)–4(c)). These findings were corroborated by the results of a real-time RT-PCR analysis (up to 1.8- and 1.3-fold difference with rAAV-h*sox9*/AlgPH155 and rAAV-hTGF-*ß*/AlgPH155 relative to rAAV-*lacZ*/AlgPH155, respectively, *P* ≤ 0.0022) (Figure 4(d)).

The administration of rAAV-h*sox9*/AlgPH155 or rAAV-hTGF-*ß*/AlgPH155 significantly decreased the levels of hypertrophic type-X collagen deposition in hMSCs after 21 days relative to rAAV-*lacZ*/AlgPH155 (up to 1.3- and 1.5-fold difference in AOD and score, respectively, with rAAV-h*sox9*/AlgPH155 relative to rAAV-*lacZ*/AlgPH155, *P* ≤ 0.0038; up to 1.3- and 1.5-fold difference in AOD and score, respectively, with rAAV-hTGF-*ß*/AlgPH155 relative to rAAV-*lacZ*/AlgPH155, *P* ≤ 0.0071) as seen by type-X collagen immunodetection (Figures 4(e)–4(g)). These findings were corroborated by the results of a real-time RT-PCR analysis (up to 1.5- and 1.6-fold difference with rAAV-h*sox9*/AlgPH155 and rAAV-hTGF-*ß*/AlgPH155 relative to rAAV-*lacZ*/AlgPH155, respectively, *P* ≤ 0.0006) (Figure 4(h)).

Treatment with rAAV-h*sox9*/AlgPH155 or rAAV-hTGF-*ß*/AlgPH155 significantly reduced matrix mineralization in hMSCs after 21 days relative to rAAV-*lacZ*/AlgPH155 (up to 1.1- and 3-fold difference in AOD and score, respectively, with rAAV-h*sox9*/AlgPH155 relative to rAAV-*lacZ*/AlgPH155, *P* ≤ 0.0111; up to 1.2- and 3-fold difference in AOD and score, respectively, with rAAV-hTGF-*ß*/AlgPH155 relative to rAAV-*lacZ*/AlgPH155, *P* ≤ 0.0019) as seen by alizarin red staining (Figures 4(i)–4(k)).

## 4. Discussion

Therapeutic rAAV vectors are promising gene transfer systems for the treatment of traumatic articular cartilage defects and osteoarthritic lesions, especially when applied to sites of cartilage injury in a spatiotemporal manner upon release from biocompatible scaffolds like those based on alginate compounds [18]. Biomaterial-guided gene therapy might be more potent than approaches based on scaffold-free gene transfer [16] or on the sole administration of alginate [18] for translational applications. In light of our recent findings showing the ability of alginate hydrogels to allow for the effective, sustained and controlled release of a reporter (*lacZ*) rAAV gene vector [19] as well as the possibility of using rAAV/alginate hydrogel systems to deliver an insulin-like growth factor I (IGF-I) to heal, at least in part, chondral defects in a conflicting (inflammatory) environment [28], we aimed at identifying other potential factors (SOX9 and TGF-*ß*) that may be of additional benefit to stimulate the regenerative activities of hMSCs via the formulation of rAAV vectors in alginate (AlgPH155) and provide convenient off-the-shelf hydrogel-based composites that may be conveniently stored upon freeze drying [29] for cartilage repair in the future.

The present findings first reveal that the administration of the reporter rAAV-*lacZ*/AlgPH155 hydrogel system allowed for significant *lacZ* overexpression in hMSCs during the period of evaluation (up to 21 days, the longest time point examined) relative to the rAAV-h*sox9*/AlgPH155 and rAAV-hTGF-*ß*/AlgPH155 hydrogel systems, corroborating the previous work with rAAV-*lacZ*/AlgPH155 [19] or other types of guided systems (self-assembling peptide hydrogels, polymeric micelles, and carbon dots) [24] to target such cells and confirming the functionality of rAAV-*lacZ* in hMSCs [30]. The data further show that the application of the candidate rAAV-h*sox9*/AlgPH155 and rAAV-hTGF-*ß*/AlgPH155 hydrogel systems significantly enhanced the levels of SOX9 and TGF-*ß* production in hMSCs, respectively, relative to control conditions (rAAV-*lacZ*/AlgPH155 and each respective counterpart) over time (up to 21 days), probably resulting from the effective release of rAAV from AlgPH155 as previously reported when using rAAV/AlgPH155 composites [19]. This observation also confirms the functionality of rAAV-h*sox9* [31] and rAAV-hTGF-*ß* [32] in hMSCs and extends work in other types of guided systems (polymeric micelles, carbon dots) [24] to target such cells.

The results next indicate that the delivery of the rAAV-h*sox9*/AlgPH155 and rAAV-hTGF-*ß*/AlgPH155 hydrogel systems significantly and durably enhanced the biological and chondrogenic activities of hMSCs for at least 21 days relative to the rAAV-*lacZ*/AlgPH155 hydrogel system, as noted by increased levels of cell proliferation and of specific ECM marker deposition (GAGs, proteoglycans, and type-II collagen), which is in good agreement with the previous findings using rAAV-h*sox9* and rAAV-hTGF-*ß* in their free form [31, 32] and with the properties of these factors [6–8]. Interestingly, rAAV-h*sox9*/AlgPH155 was capable of triggering hMSC proliferation in contrast to findings using scaffold-free rAAV-h*sox9* [31], possibly due to a sustained, controlled gene vector release and overexpression here from the alginate-based hydrogel system [19]. Overall, rAAV-hTGF-*ß*/AlgPH155 was biologically more potent in hMSCs than rAAV-h*sox9*/AlgPH155 (higher levels of DNA and proteoglycan contents and of cell proliferation indices), at least in the experimental conditions (vector dose and time point of analysis) applied here.

The current data finally demonstrate that providing the rAAV-h*sox9*/AlgPH155 and rAAV-hTGF-*ß*/AlgPH155 hydrogel systems to hMSCs significantly and durably reduced undesirable cell hypertrophy, osteogenesis, and matrix mineralization (type-X/-I collagen deposition and alizarin red staining) for at least 21 days to a similar extent relative to the rAAV-*lacZ*/AlgPH155 hydrogel system, again concordant with the previous work conducted using scaffold-free rAAV-h*sox9* and rAAV-hTGF-*ß* [31] and with the properties of these factors [33, 34]. Interestingly, rAAV-hTGF-*ß*/AlgPH155 was capable of targeting hMSC cell hypertrophy, osteogenesis, and matrix mineralization in contrast to findings using scaffold-free rAAV-hTGF-*ß* [32], again possibly due to a sustained, controlled gene vector release and overexpression here from the alginate-based hydrogel system [19].

In summary, the present study shows the benefits of alginate-guided controlled and overexpression of therapeutic rAAV-h*sox9* and rAAV-hTGF-*ß* vectors to significantly expand a source of improved (more potent) chondroreparative cells for the purpose of cartilage repair by counterbalancing the low metabolic activities of adult hMSCs. Similar work may be attempted in a different (osteogenic) microenvironment to examine possibly different outcomes for the purpose of bone healing and/or by codelivering both gene vectors via AlgPH155 as performed in a scaffold-free manner to further expand the reparative of the therapeutic gene products as a means to promote the formation of a robust early ECM that can be retained in a durable manner in the goal of cartilage repair [20]. The value of such an off-the-shelf rAAV (*sox9*, TGF-*ß*)/alginate hydrogel system is currently being tested in articular cartilage defects in relevant, large animal models *in vivo* [28] as novel, minimally-invasive treatments for cartilage repair in a clinical scenario in the future.

## Data Availability

All data generated or analyzed during this study are included within the article.

## Supplementary Materials

Supplementary Materials Table of contents: enhanced chondroreparative activities (cell proliferation and ECM deposition) of human bone marrow-derived mesenchymal stromal cells upon rAAV-mediated *sox9* and TGF-*ß* overexpression using an alginate-based hydrogel system to control the release of the gene vectors for spatiotemporally improved clinical chondroreparative applications.
