# Supplementary material for: Effects of rAAV-Mediated Overexpression of sox9 and TGF-ß via Alginate Hydrogel-Guided Vector Delivery on the Chondroreparative Activities of Human Bone Marrow-Derived Mesenchymal Stromal Cells
Source: J Tissue Eng Regen Med. 2023 Aug 18;2023:4495697. doi: 10.1155/2023/4495697 (PMC11919174; doi:10.1155/2023/4495697)
Supplement: Supplementary Materials — Table of contents: enhanced chondroreparative activities (cell proliferation and ECM deposition) of human bone marrow-derived mesenchymal stromal cells upon rAAV-mediated sox9 and TGF-ß overexpression using an alginate-based hydrogel system to control the release of the gene vectors for spatiotemporally improved clinical chondroreparative applications. [file 4495697.f1.docx]

**TABLE OF CONTENTS**

**

**

Enhanced chondroreparative activities (cell proliferation, ECM deposition) of human bone marrow-derived mesenchymal stromal cells upon rAAV-mediated *sox9* and TGF-ß overexpression using an alginate-based hydrogel system to control the release of the gene vectors for spatiotemporally improved clinical chondroreparative applications.
